# Development of an atrazine immunoassay based on highly catalytically active Au@PtNPs and specific nanobody

**DOI:** 10.21203/rs.3.rs-7065158/v1

**Published:** 2025-07-15

**Authors:** Jia Kang, Meng Qi, Yasen Wang, Linchun Li, Xinda Zhou, Xiaoshuo An, Bruce D. Hammock, Jinlin Zhang, Jingqian Huo

**Affiliations:** Hebei Agricultural University; Langfang Normal University; Hebei Agricultural University; Hebei Agricultural University; Hebei Agricultural University; Hebei Agricultural University; University of California; Hebei Agricultural University; Hebei Agricultural University

**Keywords:** Atrazine, nanobody, Au@PtNPs, direct competitive enzyme immunization, lateral flow immunoassay

## Abstract

Atrazine, one of the mostly used herbicides globally, is characterized as long residual effects on sensitive crops such as wheat, soybeans, and sweet potatoes. Also, its prolonged residues in the environment can jeopardize the health of non-target organisms and the safety of environmental ecosystems. Therefore, it is important to develop efficient and sensitive methods to detect trace atrazine in the environment. In this study, the nanobodies specifically recognizing atrazine were successfully obtained through immunize alpaca and phage display libraries, and an indirect competitive immunoassay (ic-ELISA) based on nanobodies was constructed with an ${\mathrm{IC}}_{50}$ of 0.062 μg/mL and a minimum detection limit of 0.01 μg/mL. Meanwhile, an Au@PtNPs with excellent colorimetric performance, high catalytic activity, and high stability was synthesized. The combination of Au@PtNPs and nanobodies led to the development of a direct competitive immunoassay (dc-ELISA) with higher specificity, convenience, lower cost and an ${\mathrm{IC}}_{50}$ value of 0.032 μg/mL which was lower than the ic-ELISA. Based on the Au@PtNPs and nanobodies, a novel LFIA model for qualitative and semi-quantitative detection of atrazine was constructed with a minimum detection limit of 5 ng/mL. The present study is of great significance for the construction of atrazine immunoassay combining nanobodies and nanoenzymes, which provides a sensitive, specific, simple and reliable method for the detection of atrazine in the environment.

## 1. Introduction

Atrazine, belongs to the triazene herbicide, is one of the conventional and low-cost herbicides in China and worldwide. Atrazine is characterized as long residual effects on sensitive crops such as wheat, soybeans, and sweet potatoes [1, 2], and its residual effects in the environment on the health of nontarget organisms such as humans and the safety of the environmental ecosystems are of increasing concern [3-5]. Potential human health risks of atrazine have been shown to include congestion of the heart, lungs and kidneys, low fetal weight and disruption of hormone levels [6-8]. In addition, atrazine is poorly adsorbed in soil and is water-soluble, easily leached into the soil by rainfall and not easily degraded, with a half-life of 16–77 days in soil and 150–180 days in groundwater [9], thus causing serious contamination of groundwater and surface water. Therefore, the development of efficient and sensitive atrazine residue detection technology is essential for its immediate monitoring.

Currently, the residue detection methods for atrazine include spectrometry, electrochemical methods, immunoassay and chromatography, among which spectrometry is mainly infrared spectrometry [10], and chromatography is mainly high-performance liquid chromatography (HPLC), chromatography-mass spectrometry, ultra-high-performance liquid chromatography (UHPLC), gas chromatography (GC), and gas chromatography-mass spectrometry (GC-MS) [11-13]. Electrochemical methods mainly utilize electrochemical sensors to detect atrazine [14]. Current immunoassay techniques are mainly based on antigen-antibody reaction [15-16]. In immunoassays, previous reports were mainly based on polyclonal antibodies (pAb) and monoclonal antibodies (mAb). It often needed to spend a long time to prepare and screen monoclonal hybridoma cell lines that could produce high-titer antibodies after immunizing animals. While polyclonal antibodies were relatively simple to prepare, but they would produce a large number of non-specific antibodies, which might generate background signals in some applications, and due to their possession of multiple antigen recognition epitopes, cross reactions were easy to occur. Compared with traditional antibodies, nanobodies showed great potential in the fields of rapid detection, targeted therapy, molecular imaging and so on by virtue of their small size, high stability, low cost and engineering advantages, especially suitable for the detection and point-of-care detection of trace pollutants in complex matrices. Immunochromatography (LFIA) had became an important tool for field detection because of its advantages of rapidity, sensitivity and portability. However, traditional colloidal gold immunochromatographic test strips (LFIAs) often relied on a single passive colorimetric signal output, which had limitations such as low sensitivity, narrow detection range, susceptibility to interference, and required long incubation time due to lack of catalytic amplification, which restricted their practical application [17]. With the development of nanotechnology, nanomaterials with excellent optical and catalytic properties provided new solutions for immunodetection [18, 19]. Among them, porous gold platinum nanoenzymes (Au@PtNPs), as a new type of core-shell nanomaterials, showed significant performance advantages over traditional colloidal gold. The unique porous polycrystalline platinum shell structure of Au@PtNPs could selectively block macromolecules from entering pores and allowed small molecules to pass freely, thus realizing efficient and specific catalytic amplification in protein-rich serum environment [20]. In addition, due to the synergistic effect of gold and platinum, electron transfer effect and high specific surface area characteristics, Au@PtNPs showed far superior catalytic activity to pure platinum and other nanoenzymes [21, 22], which make it possible to completely replace the traditional HRP-TMB color system and become a more efficient signal marker in immunoassay [23]. These characteristics make Au@PtNPs overcome the shortcomings of traditional colloidal gold technology and provided a solution to improve the detection performance of LFIA.

In this study, a new enzyme immunoassay and lateral flow immunoassay technique with high specificity and good sensitivity for detection of atrazine was developed by combining the nanobodies with the nanoenzymes. The sensitivity and specificity of ELISA and lateral flow immunoassay were further improved to lay a foundation for real-time monitoring of atrazine in the environment.

## 2. Materials and methods

### 2.1 Materials

Top 10 F' receptor cells, platinum Taq DNA polymerase, bovine serum albumin (BSA), and sodium dodecyl sulfate-polyacrylamide gel electrophoresis (SDS-PAGE) gels used in this study were purchased from Thermo Fisher, USA. Co-phage M13KO7, SfiI restriction endonuclease, and T4 ligase were provided by New England Biolabs, USA. Anti-6X His tag^®^ antibody and HRP Anti-6X His tag^®^ antibody were obtained from Shanghai Abbott Anti Trading Co. 99% Trehalose was purchased from Huazhong Haiwei (Beijing) Gene Technology Co. Single-component p-TMB color development solution was obtained from Beijing Solebo Technology Co. Ascorbic acid was purchased from Aladdin Reagent (Shanghai) Co. *E. coli* ER2738 electroreceptor was purchased from Biosearch Technologies, UK. 3,3',5,5'-tetramethylbenzidine (TMB), 98% HAuCl_4_ solution, H_2_PtCl_6_.H_2_O solution, atrazine and its analogs (simazine, terbuthylazine, terbutryn, cyanuric acid, ametryn, melamine, atrazine-2-hydroxy and 2-chloro-4,6-diamino-1,3,5-triazine) were purchased from Sigma Aldrich Trading Co.

### 2.2 Immunization and antiserum assessment

Nanobody production was induced using an immunogen (2h-CON) according to standard alpaca immunization procedures. A brief immunization procedure was described below. 50.0 mL of serum was collected as a negative control prior to immunization. For the initial immunization, the amount of immunogen was 1.00 mg, which was fully emulsified with an equal volume of Fuchs' complete adjuvant in a syringe and then immunized in the alpaca. This was followed by six booster immunizations with an immunogen dosage of 0.500 mg and mixed with an equal volume of Fuchs' incomplete adjuvant. One week after the 3rd immunization, 25.0 mL of carotid artery blood was obtained from the alpacas and tested for serum potency. Meanwhile, anticoagulated blood was collected for subsequent preparation of nanobodies using ethylenediaminetetraacetic acid (EDTA) anticoagulation tubes after the fifth immunization.

### 2.3 Library construction and characterization of atrazine nanobodies

Peripheral blood lymphocytes were isolated from the collected serum and total RNA was extracted, then reverse transcribed into cDNA by reverse transcription polymerase chain reaction. Two-step PCR was used to amplify the gene fragment of nanobodies, and the first round of amplification used CALL001/CALL002 as primers, while the second round of amplification used three pairs of primers, with the upstream primers were VHH-F, and the downstream primers were R3, R4, and JH, respectively, to amplify the nanobody fragment in IgG2, IgG3b, and IgG3a, respectively. The specific sequence information of the primers was shown in Table S1. The DNA fragments were digested and ligated into the vector pComb3x, which was electrotransformed into ER2738 receptor cells, and then infected with co-phage M13KO7 to construct a phage-displayed nanobody library. After amplification, the positive candidate nanobodies screened by phage ELISA were expressed as vectors in Top 10F'. In addition, the expression conditions were optimized and the nanobodies were identified by SDS-PAGE.

### 2.4 Establishment of an ic-ELISA based on atrazine nanobodies

#### 2.4.1 Determination of the sensitivity

In this study, ic-ELISA was first used to assess the effect of two coating antigens on the sensitivity of the nanobody. The optimal concentrations of nanobody and coating antigen were confirmed by the checkerboard method, and the sensitivity of nanobody was evaluated under optimal conditions. Detailed procedures for sensitivity assessment of nanobody were presented in the Supplementary Material.

#### 2.4.2 Determination of thermal stability

In order to evaluate the thermal stability of the nanobodies, the purified nanobody Nanobody-26 was heated at different temperatures (20°C, 40°C, 60°C, 75°C and 95°C) for 5 min, while another set of treatments were set up in which the Nanobody-26 was heated at 85°C for 0, 5, 15, 25, 35, 45 and 60 min, respectively. Atrazine monoclonal antibody was subjected to the same treatment. The binding activities of the nanobody and monoclonal antibody to the coating antigen were compared to assess the stability of the nanobodies.

#### 2.4.3 Determination of specificity

Cross-reactivity evaluation was performed using the developed ic-ELISA for atrazine, simazine, terbuthylazine, terbutryn, cyanuric acid, ametryn, melamine, atrazine-2-hydroxy and 2-chloro-4,6-diamino-1,3,5-triazine. The above compounds were prepared into standard solutions at certain concentrations, and the standard inhibition curves were established. The cross-reactions were calculated according to the respective ${\mathrm{IC}}_{50}$ obtained from the standard inhibition curves and the following formula:

$$\text{Cross-reaction}\;(\text{CR}\%)={\mathrm{IC}}_{50}\;\left(\text{atrazine}\right)∕{\mathrm{IC}}_{50}\;(\text{the other structurally similar compound})$$

### 2.5 Preparation of Au@PtNPs and Au@PtNPs-nanobody probes

The specific synthesis steps of AuNPs was described in the Supporting Material. Au@PtNPs were synthesized on the basis of AuNPs using a seed-mediated growth method. Firstly, 200 μL of AuNPs was added dropwise into a glass vial containing 800 μL of deionized water, followed by the addition of 20% polyvinylpyrrolidone (PVP) solution (50 μL) dropwise at a stirring speed of 500 rpm/min, and then after mixing for 5 min, 40 μL of chloroplatinic acid (100 mM) and 40 μL of ascorbic acid (100 mg/mL) were added, and the solution was then put into a water bath at 65°C and heated for 30 min. After stirring for 30 min, when the color of the solution gradually changed from light red to black brown, indicating that the preparation of Au@PtNPs was completed. The glass vials were taken out and cooled at room temperature with stirring, and then were stored at 4°C for use. The synthesis of Au@PtNPs with five different particle sizes was realized by changing the volume amount of chloroplatinic acid (100 mM) (5 μL 10 μL, 20 μL, 40 μL and 60 μL).

The sodium chloride interference method was used to determine the optimal conditions for coupling. Adjust the Au@PtNPs-145 nm (10 mL) to the optimal pH with 0.1 M K_2_CO_3_, then the optimal amount of nanobody and BSA (0.1 g) were added dropwise to the solution and stirred for 30 minutes. PEG20000 (0.05 g) was then added to the above solution and incubated at 4°C overnight after stirring for 30 minutes. The probe was purified by fractionation and centrifugation at 4°C, and the precipitate was collected and re-dissolved in 1 mL of probe diluent (pH 7.2, PBS buffer containing 1% sucrose, 0.1% BSA, 0.1% Tween 20 and 0.1% alginate) at 4°C for storage and further use.

### 2.6 Evaluation of catalytic activity of Au@PtNPs and Au@PtNPs-nanobody

HRP and Au@PtNPs – 145 solutions were prepared at the same concentration (3 pmol/L), and 100 μL of each solution was mixed with an equal volume of H_2_O_2_-TMB at different reaction temperatures (4, 25, 37, 45, 60, 75, and 85 °C) for 5 min. The catalytic activity and tolerance of HRP and Au@PtNPs-145 at different temperatures were compared and analyzed. Au@PtNPs-145 nm and Au@PtNPs-nanobody were simultaneously reacted with H_2_O_2_-TMB at 25°C to compare the differences in their catalyticactivities. At the same time, in order to verify the stability of the Au@PtNPs, the catalytic activity under different pH conditions and its tolerance to repeated freeze-thaw cycles were evaluated. After that, the changes in catalytic activity of different concentrations of Au@PtNPs-nanobody probes were also evaluated.

### 2.7 Establishment of immnoassay based on Au@PtNPs-nanobody

#### 2.7.1 Au@PtNPs-nanobody based dc-ELISA

Au@PtNPs-nanobody was used to develop a new dc-ELISA, and the optimal concentrations of probe and coating antigen were confirmed by the checkerboard method, and the sensitivity of dc-ELISA was evaluated under optimal conditions. The cross-reactivity of the dc-ELISA to atrazine analogs was also evaluated to compare with the specificity of ic-ELISA.

#### 2.7.2 Au@PtNPs-nanobody based LFIA

A new detection mode LFIA method was developed using the peroxidase-like activity of Au@PtNPs-nanobody. The new detection mode LFIA consists of an assay test strip and a staining solution. The assay test strip consists of a sample pad, nitrocellulose (NC) membrane, absorbent pad, and base card. The sample pad was immersed in buffer (0.01 M PBS, pH 7.4, containing 1% BSA, 0.5% PEG20000, and 0.5% Tween) for 60 min at 37°C and then dried at 37°C overnight. 1 μL/cm of 2e-BSA in 0.01 M PBS (pH 7.4) was added as the test (T) line, and 1μL/cm of Anti-6X His tag^®^ antibody in 0.01 M PBS (pH 7.4) was added as the control (C) line, and then the NC membranes were dried at 37°C for 24 h. Finally, the sample pads, NC membranes, and absorbent pads were assembled sequentially on a base card and then cut into 4 mm width. Commercial pTMB solution was used as the staining solution.

Different concentrations of atrazine standard (0, 0.1, 0.5, 1, 5, 10, 25, 50, 75, 100, 200 ng/mL) were analysed by the new mode LFIA. 4 μL of Au@PtNPs-nanobody probe was added to 100 μL of each solution with different concentrations of atrazine and incubated for 5 minutes. Finally, the mixture was transferred to a sample pad and reacted for 15 min and then immersed in a color development solution for 10 min to evaluate the sensitivity.

### 2.8 Analysis of spiked samples

Tap water, river water and soil without atrazine were selected as samples for the evaluation of matrix effects. The effect of tap water, river water and soil on the sensitivity of the detection method was evaluated using two newly developed immunoassay methods based on Au@PtNPs-nanobody. The accuracy of the developed method for real samples was also verified using high performance liquid chromatography (HPLC). The solid sample (10 g) was simultaneously extracted with 20 ml of methanol in a vortex mixer for 15 minutes. The mixture was then centrifuged at 4000 rpm for 5 minutes and the supernatant was collected, diluted appropriately and analyzed by HPLC. After confirming that the samples were free of atrazine, atrazine solution was added to the samples so that the final concentrations of atrazine in the samples were 1 μg/mL, 5 μg/mL, and 10 μg/mL, and then the samples were subjected to dc-ELISA. HPLC was also used for validation.

For HPLC, the water samples (20 mL) were added to a solution of acetonitrile (40 mL) contained NaCl (15 g). Then, anhydrous sodium sulfate (5 g) was added to the above mixture. The organic phase was collected, filtered and evaporated to dryness. The soil samples (20 g) were mixed with acetonitrile (40 mL) contained NaCl (15 g) and extracted by shaking for 1 h. Then, anhydrous sodium sulfate (5 g) was added to the above mixture. Organic phase was collected and evaporated to dryness. The residues of tap water, river water and soil samples were dissolved with methanol (5 mL) and analyzed by HPLC. The detection of HPLC was performed using methanol-water (1:1, v/v) as the mobile phase at 230 nm with a flow rate of 0.5 mL/min and an injection volume of 20 μL.

## 3. Results and discussion

### 3.1 Preparation of nanobodies

**Figure 1:**
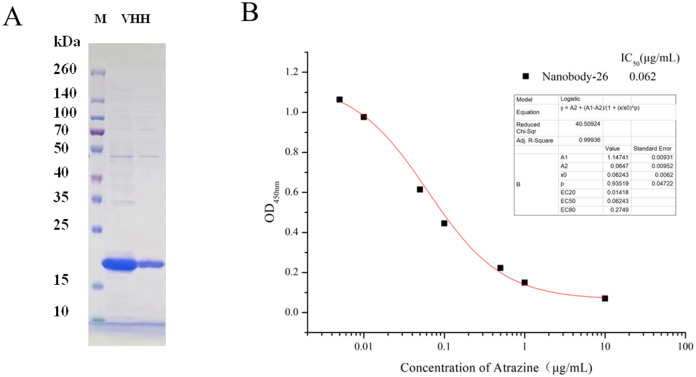
(A) SDS-PAGE images of atrazine nanobody; (B) Sensitivity evaluation of nanobody-26 against atrazine.

Starting from the third immunization, antiserum was taken one week after each immunization to measure their potency and the results (Fig S1A) showed that the titer of the antiserum increased with the strengthening of immunity. The antiserum titer no longer increased after the fifth immunization, therefore, anticoagulated blood was collected one week after the fifth immunization using EDTA anticoagulation tubes for subsequent preparation of nanobodies. The effect of the coating antigens 2e-BSA and 2e-OVA on the sensitivity of the ic-ELISA was evaluated by the checkerboard method. The best inhibition was achieved by the coating antigen 2e-BSA (Fig. S1B). Therefore, 2e-BSA was selected as coating antigen for the following studies. Peripheral blood lymphocytes were isolated from the serum of the 5th immunization to extract total RNA (Fig. S2A) for cDNA synthesis. After two rounds of PCR, the nanobody sequences were successfully amplified and ligated with the vector pComb3x and electrotransfected into ER2738 receptor cells (Fig. S2B-S2C) and further used for diversity assessment of phage display libraries, which showed good diversity. The best positive clones obtained after three rounds of phage ELISA were screened and induced by 1.0 mM of opropyl β-D-1-thiogalactopyranoside at 30°C for 16h for the prokaryotic expression of nanobodies (Figs. S3A-S3C). A clear band was identified by SDS-PAGE at around 17.5 KDa (Fig. 1A), confirming of the successfully expression of atrazine nanobodies. Nanobodies are a class of heavy chain antibodies that are different from traditional antibodies in that they are naturally missing the light chain, and they are the smallest antigen-binding fragments with complete functions that have been discovered so far [24, 25]. Nanobodies have many advantages when compared with traditional polyclonal and monoclonal antibodies, such as easy to be expressed in large quantities, good stability, and the ability to be structurally modified, etc. Nanobodies have been widely used in many fields.

### 3.2 Performance evaluation of atrazine nanobodies

An ic-ELISA based on the atrazine nanobody was constructed to evaluate the sensitivity, thermal stability and specificity of the nanobody. Based on the optimal concentration of the coating antigen and nanobody screened by the checkerboard method, a standard curve was established. The ${\mathrm{IC}}_{50}$ value of the standard inhibition curve was 0.062 μg/mL, and the lowest detection limit was 0.01 μg/mL with the linear range of 0.014 μg/mL ~ 0.275 μg/mL (Fig. 1B). According to the relevant literature, the maximum residue limits (MRLs) of atrazine in foods set by the EU [22] and the US [23] were 0.02 mg/kg to 15 mg/kg. China's latest national standard (GB2763-2021) specified MRLs of 0.05 mg/kg, 0.05 mg/kg and 0.1 mg/kg in fruits, vegetables and tea, respectively. The ic-ELISA established in this study had a minimum detection limit lower than the critical limit mentioned above (especially the common 0.02 mg/kg or 0.05 mg/kg), and its sensitivity was sufficient to meet the regulatory requirements for the detection of trace atrazine residues in real samples. Meanwhile, the thermal stability of the atrazine nanobody was characterized by comparison with atrazine monoclonal antibody previously developed and stored in our laboratory (Fig. S4A, S4B). As the temperature increases, monoclonal antibodies gradually lost their binding ability, whereas nanobodies were still able to bind to the coating antigen at temperatures as high as 95°C. The activity of the nanobody could be maintained at about 90% after 60 min of incubation at 85°C, while the monoclonal antibody lost its activity after 15 min of incubation. The results show that the nanobodies have good thermal stability.

### 3.3 Characterization of Au@PtNPs and Au@PtNPs-nanobody probes

**Figure 2:**
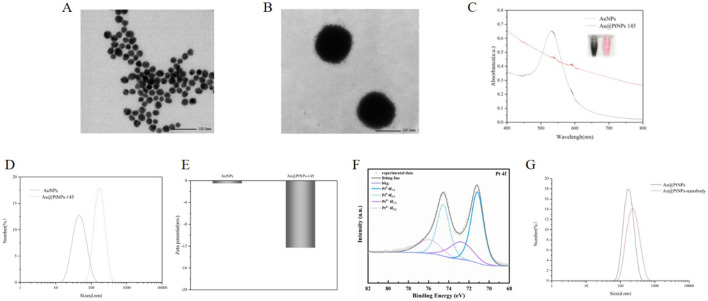
(A) TEM images of AuNPs; (B) TEM images of Au@PtNPs-145 nm; (C) UV absorption images of AuNPs and Au@PtNPs-145 nm; (D) Water-dispersed particle size of AuNPs and Au@PtNPs-145 nm; (E) Zeta potentials of AuNPs and Au@PtNPs-145 nm; (F) XPS spectra of Au@PtNPs-145 nm; (G) Water-dispersed particle size of Au@PtNPs-145 nm and Au@PtNPs-nanobody.

A seed-mediated growth method was used to synthesized Au@PtNPs. The transmission electron microscopy (TEM) images showed that the gold seeds exhibited a distinct spherical morphology with a smooth surface and an average diameter of 15 nm (Fig. 2A). In order to generate porous Pt layers on spherical gold seeds, polyvinylpyrrolidone (PVP) and L-ascorbic acid were used as a stabilizer and a reducing agent respectively for the growth of Pt particles. Five different Au@PtNPs with particle sizes of 70 nm, 95 nm, 105 nm, 135 nm, 145 nm, and 165 nm were synthesized (Fig. S5, Fig.S6A). Their catalytic activities were evaluated by reacting them with the same amount of H_2_O_2_-TMB at the same concentration. The results showed that Au@PtNPs-145 nm had the best catalytic activity (Fig. S6B). As the Pt layer thickens it increased the specific surface volume thereby enhancing the catalytic activity. However, too large a particle size would also affect its stability and made it easy to precipitate, thus reducing the catalytic activity. The structure and morphology of Au@PtNPs-145 nm were characterized by high resolution TEM (Fig. 2B). The best catalytic activity of Au@PtNPs-145 nm was obtained as a spherical sea urchin-like structure with relatively rough surface and good homogeneity. The UV absorption pattern showed the disappearance of the characteristic peaks of the gold seeds (Fig. 2C). The hydrodynamic diameter of the gold seeds determined by simultaneous dynamic light scattering (DLS) analysis was in agreement with the TMB (Fig. 2D). The zeta potential of Au@Pt NPs was reduced to −12.3 mV compared to AuNPs (Fig. 2E). The valence changes of Pt in Au@PtNPs were analyzed by X-ray photoelectron spectroscopy spectroscopy (Fig. 2F). The electronic binding energy on the Pt 4f orbitals of Au@PtNPs could be decomposed into two pairs of double states, Pt 4f^7/2^ and Pt 4f^5/2^, for both Pt^0^ and Pt^2+^ valence states. Among them, the presence of Pt0 was crucial for the catalytic activity of Au@PtNPs due to the unsaturated chemical bonds on its surface forming more available active sites. Energy dispersive spectroscopy (EDS) maps showed an ordered distribution of gold and platinum throughout the nanoparticles, confirming the composition of the bimetallic nanoparticles (Fig S7). A physisorption-based detection probe (Au@PtNPs-nanobody) was also prepared based on Au@PtNPs. The water-dispersible particle size of the atrazine nanobody significantly increased after coupling with Au@PtNPs (Fig. 2G).

The probes were prepared by adding 5 μg of nanobody to 1 mL of Au@PtNPs and incubating for 20 minutes, then adding different amounts of potassium carbonate and comparing the absorbance changes before and after the addition to determine the optimal pH for coupling. Similarly, the optimal amount of antibody coupled to Au@PtNPs could be determined by adding different amounts of nanobodies to 1 mL of Au@PtNPs at the optimal pH and comparing the change in absorbance. The results showed that the optimal pH for coupling was 7.6 and the optimal amount of antibody coupled to 1 mL of Au@PtNPs was 8 μg (Fig. S8).

### 3.4 Validation of catalytic activity of Au@PtNPs and Au@PtNPs-nanobody

**Figure 3:**
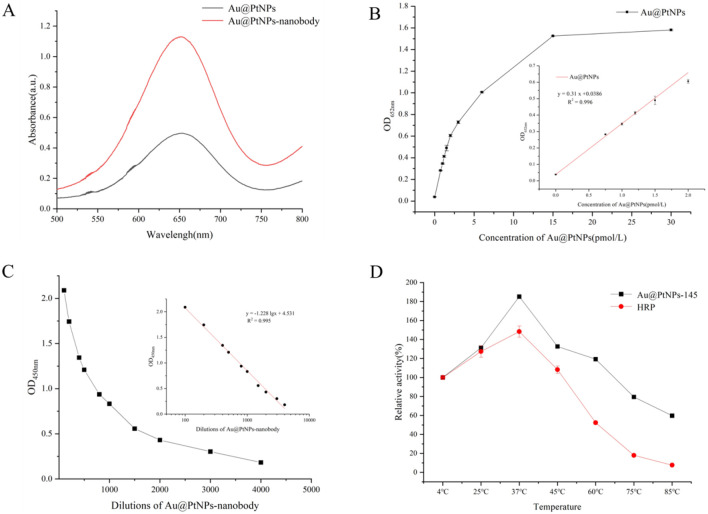
(A) UV absorption spectra of Au@PtNPs-145 nm and Au@PtNPs-nanobody at different wavelengths; (B) Catalytic activity of Au@PtNPs-145 nm; (C) Catalytic activity of Au@PtNPs-nanobody; (D) Comparison of thermal stability between Au@PtNPs-145 nm and HRP.

To verify the feasibility of Au@PtNPs as a label in ELISA and test strips, its catalytic ability was assessed. The UV absorption spectra showed the consistency of the catalytic activities of Au@PtNPs and Au@PtNPs-nanobody (Fig. 3A), and then their peroxidase activities were evaluated by controlling the substrate concentration and varying the addition of Au@PtNPs and Au@PtNPs-nanobody. The absorbance gradually increased and reached equilibrium with the increase of the concentration of Au@PtNPs-145 nm and Au@PtNPs-nanobody. Both Au@PtNPs-145 nm and Au@PtNPs-nanobody probes showed a good linear correlation with the absorbance (R^2^ = 0.996, R^2^ = 0.995), which demonstrating their good catalytic activity (Fig. 3B,3C). At the same time, compared with the traditional HRP tag, the Au@PtNPs exhibits higher thermal stability and can maintain good peroxidase activity in high-temperature environments (Fig. 3D). Under strong acid conditions, the activity of both was significantly inhibited. However, under strong alkali conditions, the activity of Au@PtNPs decreased less than that of HRP (Fig. S9A). In addition, the freeze-thaw cycle led to a sharp decrease in the activity of both Au@PtNPs and HRP, but the decay of the Au@PtNPs was relatively slow in the early stage (Fig. S9B). Based on the above results, the Au@PtNPs nanozymes synthesized in this study showed the advantages of improved overall stability compared with traditional HRP.

### 3.5 Sensitivity of the dc-ELISA based on the Au@PtNPs-nanobody probe

The peroxidase-like activity of Au@PtNPs catalyzes the production of deeper color signals from PTMBs, which greatly enhances their intrinsic optical properties. The catalytic color signal combined with the intrinsic color of the label can significantly enhance the signal intensity, which can greatly improve the detection range and sensitivity of small molecules. Therefore, novel dc-ELISA and LFIA assays were developed using Au@PtNPs conjugated to nanobodies as labels, which not only saved cost and made the assay more convenient, but also improved the sensitivity and specificity of nanobodies.

**Figure 4:**
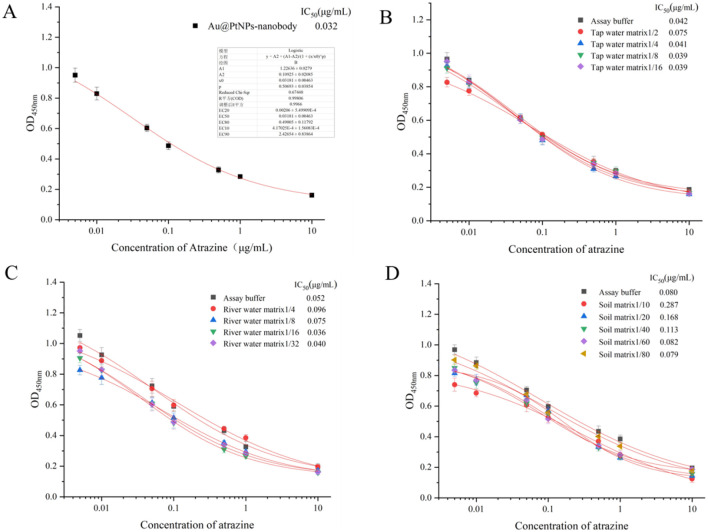
(A) Sensitivity of Au@PtNPs-nanobody; (B) Evaluation of the tap water matrix effect on dc-ELISA assay; (C) Evaluation of the river water matrix effect on dc-ELISA assay; (D) Evaluation of the soil matrix effect on dc-ELISA assay.

For the dc-ELISA, the optimal concentrations of the coating antigen 2e-BSA and the Au@PtNPs-nanobody probe were first determined by the checkerboard method. The highest inhibition effect was achieved when the Au@PtNPs-nanobody probe was diluted 50-fold and the concentration of 2e-BSA was 0.006 mg/mL (Fig. S10A). The effect of the BSA concentration in the closure step on the sensitivity of the nanobody was aslo evaluated and the best results were obtained when the BSA concentration was 1% (Fig. S10B). The effects of different Na^+^ concentrations and different methanol concentrations in the buffer on the sensitivity of the assay were also evaluated. The results showed that the sensitivity of the detection method decreased with the increase of both Na^+^ concentration and methanol concentration. Therefore the optimal buffer for this assay was 1×PBS buffer containing 5% methanol (Fig. S10C, S10D). The sensitivity of the Au@PtNPs-nanobody probe was evaluated under the optimal conditions (Fig. 4A). The ${\mathrm{IC}}_{50}$ value and the lowest detection limit of the developed dc-ELISA was 0.032 μg/mL and 0.002 μg/mL respectively, with the linear detection ranges of 0.002 ~ 0.49 μg/mL. The sensitivity had increased by nearly twice compared to the ic-ELISA established with a single nanobody.

**Figure 5:**
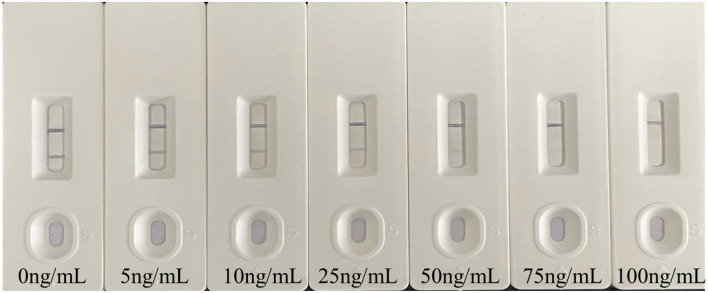
Sensitivity of LFIA method based on Au@PtNPs-nanobody (the concentration of atrazine from left to right was 0, 5, 10, 25, 50, 75, 100 ng/mL).

For a novel colorimetric LFIA based on Au@PtNPs-nanobody probe, in the absence of atrazine (negative sample), Au@PtNPs-nanobody binds to 2e-BSA immobilized on the T-line via antigen-antibody interactions, producing a black band. In the presence of atrazine (positive sample), free atrazine competes with 2e-BSA for binding to Au@PtNPs-nanobody, resulting in a decrease in the amount of Au@PtNPs-nanobody bound to the T-line. As the concentration of atrazine increased, the black band on the T-line gradually disappeared. Finally, when the test strip was immersed in the color development solution, the color signal generated by catalysis was superimposed on the T-line, and the signal intensity increased, which greatly improved the detection performance. The color of the T-line was observed by naked eye for qualitative analysis. The sensitivity of the Au@PtNPs-nanobody probe was evaluated using a new model LFIA assay with different concentrations of atrazine standard solutions (Fig. 5). When the concentration of atrazine was 5 ng/mL, the color of the T-line started to lighten and could be distinguished from the negative control. Therefore, 5 ng/mL was considered to be the lowest detection limit of atrazine in the test strip. When the atrazine concentration reached 100 ng/mL, the color at the T-line disappeared. Therefore, the results of atrazine detection by the test strips were categorized into three types: (1) negative (−), the concentration of atrazine in the samples was < 5 ng/mL; (2) weakly positive (±), the concentration of atrazine in the samples ranged from 5 to 75 ng/mL; (3) positive (+), the concentration of atrazine in the samples was > 100 ng/mL.

### 3.6 Specificity of the dc-ELISA based on the Au@PtNPs-nanobody probe

The specificity of single nanobody was first evaluated based on the developed ic-ELISA method using structural analogs of atrazine. The results (Table 1) showed that the cross-reactivity of the nanobody with terbutryn was greater than 70%, whereas the cross-reactivity with simazine and ametryn was about 30%. Therefore, the specificity of the nanobodies could be improved. In order to further improve the specificity of nanobodies, Au@PtNPs-nanobody-based dc-ELISA and LFIA methods were also established.

**Figure 6:**
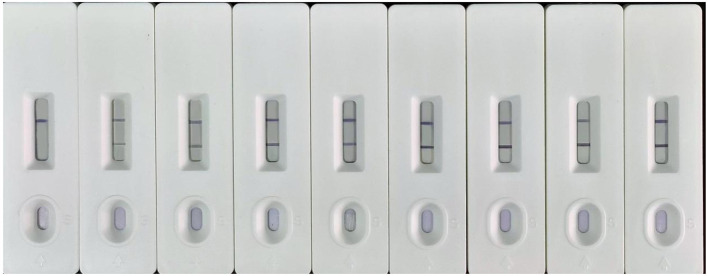
Specificity of the LFIA method based on Au@PtNPs-nanobody (the concentration of the compound was 100 ng/mL, and the order from left to right was atrazine, terbuthylazine, simazine, ametryn, 2-chloro-4,6-diamino-1,3,5-triazine, cyanuric acid, terbutryn, and melamine).

For the dc-ELISA, the specificity of the nanobody after coupling Au@PtNPs was greatly improved, with cross-reactivity greater than 10% only with terbutryn, and less than 7% for both simazine and deisopropylatrazine (Table 1). It is speculated that the nanobody coupled to Au@PtNPs hindered the binding sites of the nanobody to the atrazine analogs, leading to a much higher specificity. The newly established LFIA was aslo used to further validate the specificity of the Au@PtNPs-nanobody probe using structural analogs of atrazine (at a concentration of 100 ng/mL). The results showed that the Au@PtNPs-nanobody probe in the LFIA method presented a specificity that was essentially the same as that of the dc-ELISA (Fig. 6).

### 3.7 Validation of matrix effects and spiking-recovery studies

Considering the real samples detection, complex matrix can affect the recognition and binding between antibodies and coating antigens/analytes, which seriously interferes with the performance of the immunoassay and affects the accuracy of the analytical results. Therefore, matrix effects should be minimized or eliminated during sample analysis to ensure the accuracy of the immunoassay method. The simplest method for matrix elimination or reduction is to dilute the sample with the assay buffer. In this study, tap water, river water and soil were selected for matrix effect evaluation. The tap water samples confirmed to be none of atrazine were diluted directly with 1×PBS to a total dilution ratio of 2-, 4-, 8-, and 16-fold, and then different concentrations of atrazine solution were prepared with these obtained buffer solutions, respectively. The results (Fig. 4B) displayed that there was no significant difference in maximum absorbance under different dilution ratios of tap water. But, tap water samples diluted 4-fold had higher ${\mathrm{IC}}_{50}$ when compared with assay buffer or other diluted samples. When the tap water samples were diluted 4-, 8- and 16-fold, there was no significant difference in maximum absorbance and ${\mathrm{IC}}_{50}$ when compared with assay buffer. Therefore, the 4-fold dilution was selected for the tap water samples. Similarly, river water samples diluted 4-fold and 8-fold had higher ${\mathrm{IC}}_{50}$ values compared to assay buffer or other diluted samples. When river water samples were diluted 16-fold, 32-fold and there was no significant difference in the maximum absorbance and ${\mathrm{IC}}_{50}$ compared to the assay buffer. Therefore, 16-fold dilution was selected for the river water sample (Fig. 4C). Soil samples without atrazine were used as blank samples and extracted with methanol, then diluted with 1×PBS resulting in a total dilution ratio of 10, 20, 40, 60 and 80-fold. Atrazine standard solutions were prepared using the above obtained buffer solutions. The results (Fig. 4D) showed that the soil samples diluted 10, 20, and 40-fold had the lower maximum absorbance and higher ${\mathrm{IC}}_{50}$ than those of other dilutions. When the soil matrix was diluted 60 and 80-fold, there was no significant difference between their maximum absorbance and ${\mathrm{IC}}_{50}$. Therefore, the 60-fold dilution was selected for the next spike and recovery study. Spiked-recovery validation using both HPLC and dc-ELISA to evaluate the precision of the dc-ELISA. The results showed that (Table 2) the average recoveries ranged from 97.4–99.1% for tap water, 100.4–103% for river water samples, and 99.4–114% for soil samples analyzed by dc-ELISA. Also, the results of the dc-ELISA method were approximately consistent with that of HPLC. These results indicate that the Au@PtNPs-nanobody-based dc-ELISA developed in this study could be used for the detection of atrazine residues in tap water, river water and soil with acceptable accuracy and reproducibility.

For the LFIA, the extracted matrix solution was diluted to 4, 8 and 16 fold, and then different concentrations of atrazine (0, 10, 25 and 50 ng/mL) were prepared for testing, and the results were compared with those of the matrix-free solution (CK) to examine the effect of matrix on the accuracy of the test strips (Fig. S11A, S11B, S11C). The matrix effect was basically eliminated after 4-fold, 8-fold and 16-fold dilutions of tap water, river water and soil samples. Spiked-recovery studies were also carried out in three matrixes using LFIA (Table S2). Tap water, river water and soil samples without atrazine were spiked with different concentrations of atrazine to give final atrazine concentrations of 0, 20, 50 and 100 ng/g. For the tap water, the samples needed to be diluted 4 times before testing. When the final concentration of atrazine was > 20 ng/g in tap water samples, the test strip showed positive. While when the final concentration of atrazine in tap water samples was < 20 ng/g, the test paper was negative. The river water samples needed to be diluted 8 times before testing. The test strip was positive with a final concentration of atrazine in river water samples was > 50 ng/g. When the final concentration of atrazine in river water samples was < 50 ng/g, the test strip was negative. After diluting the samples 16 times, the test strips were positive for atrazine in soil samples with a final concentration of > 100 ng/g. When the final concentration of atrazine in soil samples was < 100 ng/g, the test strip was negative. Although the new model LFIA had great potential to improve the detection performance of the traditional LFIA, there were still several issues that deserve further consideration for practical applications. First, these properties, such as fluorescence quenching and photothermal effects of Au@PtNPs and Raman scattering, still need to be further explored in order to develop multimodal LFIAs that could be applied in different scenarios for detection.

In conclusion, a novel nanobody against atrazine was obtained through alpaca immunization, serum evaluation, phage display library construction, affinity panning, and an ic-ELISA was successfully developed to detect atrazine with an IC50 value of 0.062 μg/mL. In order to improve the sensitivity and specificity of nanobody and to apply it to a wider range of scenarios, an Au@PtNPs with excellent colorimetric properties, high catalytic activity and stability was synthesized and a novel dc-ELISA with higher specificity, good sensitivity, greater convenience, and lower cost was developed by combining Au@PtNPs and nanobodies with the IC50 value was 0.032 μg/mL. Also, a novel LFIA model for qualitative and semi-quantitative detection of atrazine was constructed based on Au@PtNPs and nanobodies with a minimum detection limit of 5 ng/mL. This work demonstrated the great potential of Au@PtNPs as multifunctional labels in immunoassay methods and opened a new avenue for the development of high-performance immunoassay methods for the detection of trace analytes.

## 4. Conclusion

In this study, a novel nanobody against atrazine was obtained through alpaca immunization, serum evaluation, phage display library construction, affinity panning, and an ic-ELISA was successfully developed to detect atrazine with an ${\mathrm{IC}}_{50}$ value of 0.062 μg/mL. In order to improve the sensitivity and specificity of nanobody and to apply it to a wider range of scenarios, an Au@PtNPs with excellent colorimetric properties, high catalytic activity and stability was synthesized and a novel dc-ELISA with higher specificity, good sensitivity, greater convenience, and lower cost was developed by combining Au@PtNPs and nanobodies with the ${\mathrm{IC}}_{50}$ value was 0.032 μg/mL. Also, a novel LFIA model for qualitative and semi-quantitative detection of atrazine was constructed based on Au@PtNPs and nanobodies with a minimum detection limit of 5 ng/mL. This work demonstrated the great potential of Au@PtNPs as multifunctional labels in immunoassay methods and opened a new avenue for the development of high-performance immunoassay methods for the detection of trace analytes.

## Supplementary Material

Nanobody-based ic-ELISA; Synthesis of AuNPs; Au@PtNPs-nanobody probe-based dc-ELISA; Information of primers for nanobody gene amplification and sequencing; Results of the spike and recovery study based on test strips; Titers of serum; Effect of different concentrations of 2e-BSA, 2e-OVA and antibody on the sensitivity of ic-ELISA; Construction of phage-displayed nanobody libraries; Optimization of nanobody expression conditions; Comparison of thermal stability between nanobody and monoclonal antibody; TEM images of Au@PtNPs with different particle sizes; The performance of Au@PtNPs with different particle sizes; The EDS mapping of AuNPs and Au@PtNPs, EDS energy spectrum of Au@PtNPs; Optimization of coupling conditions for Au@PtNPs and nanobodies; Stability comparison of Au@PtNPs and HRP; Optimization of dc-ELISA conditions based on Au@PtNPs-nanobody probes; Effect of different matrix on the detection sensitivity of test strips.

This is a list of supplementary files associated with this preprint. Click to download.

Supportinginformation.docx

supplementaryfile.docx

Table1.docx

Graphicalabstract.png

Table 1

Table 1 is available in the Supplementary Files section.

## Figures and Tables

**Table 1:** Specificity of nanobody and Au@PtNPs-nanobody probes.

Compound	Structure	Nanobody		Au@PtNPs-nanobody	
		${\mathrm{IC}}_{50}(\mu g∕\mathrm{mL})$	CR%	${\mathrm{IC}}_{50}(\mu g∕\mathrm{mL})$	CR%
Atrazine		0.062	100	0.032	100
Simazine		0.18	33.6	0.53	6.01
Terbuthylazine		0.085	72.7	0.29	10.9
Terbutryn		> 100	< 0.1	> 100	< 0.1
Cyanuric acid		> 100	< 0.1	> 100	< 0.1
Ametryn		0.23	27.4	0.90	3.55
2-chloro-4,6-diamino-1,3,5-triazine		1.1	5.40	0.93	3.42
Melamine		> 100	< 0.1	> 100	< 0.1
Atrazine-2-hydroxy		> 100	< 0.1	> 100	< 0.1

**Table 2:** Spike-recovery results of three samples determined by ELISA and HPLC.

Samples	Spiked concentration(μg/mL)	Found (μg/mL)		Average recovery (%)		CV (%)	
		dc-ELISA	HPLC	dc-ELISA	HPLC	dc-ELISA	HPLC
Tap water	1	0.982 ± 0.034	1.06 ± 0.013	98.2	106	1.33	2.17
	5	4.87 ± 0.045	4.94 ± 0.038	97.4	98.8	2.09	3.21
	10	9.91 ± 0.037	10.0 ± 0.042	99.1	100	4.21	1.44
River water	1	1.03 ± 0.023	1.04 ± 0.047	103	104	1.70	2.23
	5	5.11 ± 0.043	5.04 ± 0.031	102	101	3.57	3.15
	10	10.0 ± 0.034	10.1 ± 0.028	100	101	3.05	1.98
Soil	1	1.14 ± 0.047	1.03 ± 0.052	114	103	2.63	1.74
	5	4.97 ± 0.049	5.20 ± 0.021	99.4	104	0.560	1.14
	10	10.1 ± 0.030	10.1 ± 0.036	101	101	4.76	2.42

## References

[1] Chen Y.K., Jiang Z., Wu D., Wang H.L., Li J.J., Bi M.C., Zhang Y., Development of a novel bio-organic fertilizer for the removal of atrazine in soil, J. Environ. Manage. 233 (2019) 553–560. 10.1016/j.jenvman.2018.12.086. 30597348

[2] Xie J., Guo Y., Ma Y., Jiang H., Zhang L., Mao L., Zhu L., Wu C., Zheng Y., Liu X., Biochar prevents soybean seedling injury caused by atrazine residue by regulating the concentration of this herbicide in soil pore water, Bcha. 6(1) (2024) 59. 10.1007/s42773-024-00351-0.

[3] Deng S., Chen C., Wang Y., Liu S., Zhao J., Cao B., Jiang D., Jiang Z., Zhang Y., Advances in understanding and mitigating Atrazine's environmental and health impact: A comprehensive review, J. Environ. Manage. 365 (2024) 121530. 10.1016/j.jenvman.2024.121530. 38905799

[4] Liu Z., Han L., Zhang X., Chen S., Wang X., Fang H., Core bacteria carrying the genes associated with the degradation of atrazine in different soils, Environment Int. 181 (2023) 108303. 10.1016/j.envint.2023.108303. 37948867

[5] Gao T., Tian H., Xiang L., Wang Z., Fu Y., Shi J., Wen X., Jiang X., He W., Hashsham S.A., Wang F., Characteristics of bacterial community and extracellular enzymes in response to atrazine application in black soil, Environ. Pollut. 343 (2024) 123286. 10.1016/j.envpol.2023.123286. 38171425

[6] Yang Y.-F., Cheng S.-Y., Wang Y.-L., Yue Z.-P., Yu Y.-X., Chen Y.-Z., Wang W.-K., Xu Z.-R., Qi Z.-Q., Liu Y., Accumulated inflammation and fibrosis participate in atrazine induced ovary toxicity in mice, Environ. Pollut. 360 (2024) 124672. 10.1016/j.envpol.2024.124672. 39103034

[7] Xuyan Z., Huan L., Sai Y., Ti Y., Fangda F., Ming Y., Hongfeng R., Atrazine exposure promotes cardiomyocyte pyroptosis to exacerbate cardiotoxicity by activating NF-kappaB pathway, Sci. Total Environ. 915 (2024) 170028–170028. 10.1016/j.scitotenv.2024.170028. 38224882

[8] Krishnapura S.R., McNeer E., Dupont W.D., Patrick S.W., County-Level Atrazine Use and Gastroschisis, JAMA Netw. Open 7(5) (2024) e2410056–e2410056. 10.1001/jamanetworkopen.2024.10056. 38709530 PMC11074809

[9] Singh R.P., Yadav R., Pandey V., Singh A., Singh M., Shanker K., Khare P., Effect of biochar on soil microbial community, dissipation and uptake of chlorpyrifos and atrazine, Bcha. 6(1) (2024) 17. 10.1007/s42773-024-00306-5.

[10] Trindade F.C.S., Sobrinha I.G.d.S., Pereira G., Pereira G.A.L., Raimundo I.M. Jr, Pereira C.F., A surface-enhanced infrared absorption spectroscopy (SEIRA) multivariate approach for atrazine detection, Spectrochim. Acta A 322 (2024) 124867. 10.1016/j.saa.2024.124867. 39059263

[11] Cuifang Z., Zhuang W., Sheng L., Huihua T., Dongqiang Z., Xuesheng L., Analytical method for sequential determination of persistent herbicides and their metabolites in fish tissues by UPLC-MS/MS, Chemosphere 288(Part 2) (2022) 132591–132591. 10.1016/j.chemosphere.2021.132591. 34662632

[12] Shah J., Jan M.R., Ara B., F.-u.-N. Shehzad, Quantification of triazine herbicides in soil by microwave-assisted extraction and high-performance liquid chromatography, Environ. Monit. Assess. 178(1-4) (2011) 111–119. 10.1007/s10661-010-1676-0. 20824333

[13] Skaggs C.S., Logue B.A., Ultratrace analysis of atrazine in soil using Ice Concentration Linked with Extractive Stirrer and High Performance Liquid Chromatography-Tandem Mass Spectrometry, J. Chromatogr. A 1635 (2021) 461753. 10.1016/j.chroma.2020.461753. 33285417

[14] Shu H., Lai T., Yao B., Li M., Li H., Wang S., Chen T., Xiao X., Wang Y., Synergistic effect between pn heterojunction and oxygen vacancies of Co3O4-C/Fe-MOF for highly sensitive detection of trace atrazine, Chem. Eng. J. 490 (2024) 151652.

[15] Na Y., Sheng W., Yuan M., Li L., Liu B., Enzyme-linked immunosorbent assay and immunochromatographic strip for rapid detection of atrazine in water samples, Mikrochim. Acta 177 (2012) 177–184. 10.1007/s00604-012-0772-y

[16] Sun T., Xu Z., Yuan S., Liu X., Chen Z., Han Z., Liu W., Fan L., Yang H., Qie Z., Ning B., A gold nanoparticle-based lateral flow immunoassay for atrazine point-of-care detection using a handhold scanning device as reader, Microchim. Acta 189(4) (2022)153. 10.1007/s00604-021-05146-9. 35322310

[17] Wang Z., Zou R., Yi J., Wang Y., Hu H., Qi C., Lai W., Guo Y., Xianyu Y., "Four-In-One" Multifunctional Dandelion-Like Gold@platinum Nanoparticles-Driven Multimodal Lateral Flow Immunoassay, Small 20(29) (2024) 2310869. 10.1002/smll.202310869. 38363059

[18] Atta S., Zhao Y., Sanchez S., Seedial D., Devadhasan J.P., Summers A.J., Gates-Hollingsworth M.A., Pflughoeft K.J., Gu J., Montgomery D.C., AuCoin D.P., Zenhausern F., Vo-Dinh T., Plasmonic-Enhanced Colorimetric Lateral Flow Immunoassays Using Bimetallic Silver-Coated Gold Nanostars, Acs Appl. Mater. Interfaces 16(40) (2024) 54907–54918. 10.1021/acsami.4c13086. 39342509

[19] Meng X., Zuo W., Wu P., Song Y., Yang G.-J., Zhang S., Yang J., Zou X., Wei W., Zhang D., Dai J., Ju Y., Bimetallic Nanozyme: A Credible Tag for In Situ-Catalyzed Reporter Deposition in the Lateral Flow Immunoassay for Ultrasensitive Cancer Diagnosis, Nano Lett. 24(1) (2023) 51–60. 10.1021/acs.nanolett.3c03118. 37823474

[20] Loynachan C.N., Thomas M.R., Gray E.R., Richards D.A., Kim J., Miller B.S., Brookes J.C., Agarwal S., Chudasama V., McKendry R.A., Stevens M.M., Platinum Nanocatalyst Amplification: Redefining the Gold Standard for Lateral Flow Immunoassays with Ultrabroad Dynamic Range, Acs Nano 12(1) (2018) 279–288. 10.1021/acsnano.7b06229. 29215864 PMC5785759

[21] Tang M., Zhang Z., Ding C., Li J., Shi Y., Sun T., Chen C., Two birds with one stone: innovative ceria-loaded gold@platinum nanospheres for photothermal-catalytic therapy of tumors, J. Colloid Interf. Sci. 627 (2022) 299–307. 10.1016/j.jcis.2022.07.065. 35863189

[22] Zhang M., Guo X., Gold/platinum bimetallic nanomaterials for immunoassay and immunosensing, Coordin. Chem. Rev. 465 (2022) 214578. 10.1016/j.ccr.2022.214578.

[23] Peiyuan C., Rongzhi W., Sumei L., Shihua W., A high sensitive platinum-modified colloidal gold immunoassay for tenuazonic acid detection based on monoclonal IgG, Food Chem. 360 (2021) 130021–130021. 10.1016/j.foodchem.2021.130021. 33991976

[24] Zhang J.-r., Wang Y., Dong J.-x., Yang J.-y., Zhang Y.-q., Wang F., Si R., Xu Z.-l., Wang H., Xiao Z.-l., Shen Y.-d., Development of a Simple Pretreatment Immunoassay Based on an Organic Solvent-Tolerant Nanobody for the Detection of Carbofuran in Vegetable and Fruit Samples, Biomolecules 9(10) (2019) 576. 10.3390/biom9100576. 31591300 PMC6843801

[25] Koch-Nolte F., Nanobody-based heavy chain antibodies and chimeric antibodies, Immunol. Rev. (2024) 13385. 10.1111/imr.13385. PMC11659929 39212236

